# Fetal nuchal edema and developmental anomalies caused by gene mutations in mice

**DOI:** 10.3389/fcell.2022.949013

**Published:** 2022-08-30

**Authors:** Akira Sugiyama, Masanori Hirashima

**Affiliations:** Division of Pharmacology, Graduate School of Medical and Dental Sciences, Niigata University, Niigata, Japan

**Keywords:** fetal nuchal edema, gene mutations, lymphatic vascular development, cardiac anomaly, mouse embryos

## Abstract

Fetal nuchal edema, a subcutaneous accumulation of extracellular fluid in the fetal neck, is detected as increased nuchal translucency (NT) by ultrasonography in the first trimester of pregnancy. It has been demonstrated that increased NT is associated with chromosomal anomalies and genetic syndromes accompanied with fetal malformations such as defective lymphatic vascular development, cardiac anomalies, anemia, and a wide range of other fetal anomalies. However, in many clinical cases of increased NT, causative genes, pathogenesis and prognosis have not been elucidated in humans. On the other hand, a large number of gene mutations have been reported to induce fetal nuchal edema in mouse models. Here, we review the relationship between the gene mutants causing fetal nuchal edema with defective lymphatic vascular development, cardiac anomalies, anemia and blood vascular endothelial barrier anomalies in mice. Moreover, we discuss how studies using gene mutant mouse models will be useful in developing diagnostic method and predicting prognosis.

## Introduction

In the first trimester of pregnancy, fetal nuchal edema, a subcutaneous accumulation of extracellular fluid in the fetal neck, is visualized by ultrasonography as increased nuchal translucency (NT) (Nicolaides et al., 1992). It has been demonstrated that increased NT is associated with chromosomal anomalies such as trisomy 21 (Down syndrome), trisomy 18 and trisomy 13 (Nicolaides et al., 1992; Snijders et al., 1998). In addition, increased NT in fetuses with normal karyotype is associated with defective lymphatic vascular development, cardiac anomalies, hereditary anemia, and a wide range of other fetal anomalies, including skeletal defects, central nervous system defects and diaphragmatic hernia (Tercanli et al., 2001; Souka et al., 2002; Atzei et al., 2005; Souka et al., 2005). Enhanced blood vascular permeability may also be associated with increased NT as seen in subcutaneous edema in adults (Claesson-Welsh et al., 2021). The causative genes have been identified in some but not many clinical cases of increased NT. Many cases of increased NT disappear during the second trimester and the majority of fetuses with increased NT are born normally (Yoshida et al., 2008). Therefore, increased NT is considered to be a transient physiological finding, and the prognosis of fetal nuchal edema has not been sufficiently investigated. However, fetuses with increased NT sometimes have edema worsened to develop hydrops fetalis (Tahmasebpour et al., 2012). It would be desirable to develop the clinical diagnosis to distinguish between cases that ends in temporary changes and life-threatening problems. For this purpose, it is necessary to identify additional causative genes and to understand the relationship between gene mutations, pathogenesis and prognosis.

**FIGURE 1:**
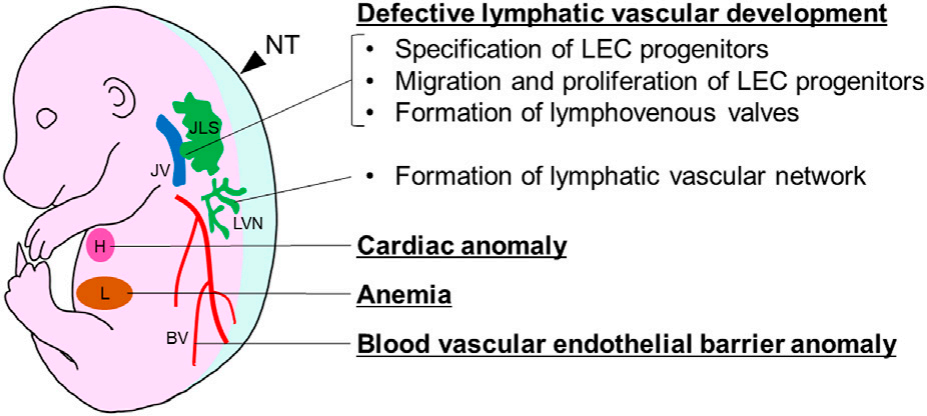
Developmental anomalies causing fetal nuchal edema in mice. BV, blood vessel; H, heart; JLS, jugular lymph sac; JV, jugular vein; L, liver; LVN, lymphatic vascular network; NT, nuchal translucency.

Compared to studies in humans, a larger number of gene mutations have been reported to induce fetal nuchal edema in mouse models. Therefore, a better understanding of the relationship between causative gene mutations and fetal phenotypes in mutant mice may provide new strategies for treatment of human clinical cases. Here, we review the relationship between the gene mutations causing fetal nuchal edema with defective lymphatic vascular development, cardiac anomalies, anemia and changes in blood vascular endothelial barrier in mice (Figure 1). Moreover, we discuss how studies using gene mutant mice will be useful in developing diagnostic method and predicting prognosis.

## Defective lymphatic vascular development

Lymphatic vessels form a network throughout the body and play important roles in tissue fluid homeostasis by collecting excess interstitial fluid and returning it to the blood circulation (Tammela and Alitalo, 2010; Yang and Oliver, 2014; Mäkinen et al., 2021). Therefore, lymphatic vascular dysfunction is a major cause of edema (Souka et al., 2005). Here, we describe stepwise processes of lymphatic vascular development by pointing out gene mutations causing fetal nuchal edema in mice (Table 1).

**TABLE 1:** List of genes and mutant phenotypes related to fetal nuchal edema with defective lymphatic vascular development in mice (loss-of-function mutations if not stated).

Gene	Mutant phenotype	References
*Prox1*	Absence of LECs and lymphatic vessels	Wigle and Oliver, 1999; Harvey et al., 2005
	(Gain-of-function) Enlarge lymph sacs	Kim et al., 2013
*Sox18*	Reduced number of LEC progenitors in the JV; narrowed lymphatic vessels with increased branching	François et al., 2008
	Hypotrichosis-lymphedema-telangiectasia syndrome (human)	
*Nr2f2*	Enlarged and blood-filled lymph sacs; reduced number of LEC progenitors in and around the JV; reduced number of lymphatic vessels; dilated lymphatic vessels with decreased branching	Lin et al., 2010; Srinivasan et al., 2010
*Hhex*	Blood-filled lymphatic vessels; reduced number of LEC progenitors in the JV; reduced density of lymphatic vessels	Gauvrit et al., 2018
*Cpt1a*	Small lymph sacs; reduced number of LEC progenitors in the JV; dilated and blood-filled lymphatic vessels with decreased branching; delayed elongation of lymphatic vessels	Wong et al., 2017
*Vegfc*	Absence of lymph sacs and lymphatic vessels; defective migration of LEC progenitors from the JV	Karkkainen et al., 2004
*Flt4*	Absence of lymph sacs and lymphatic vessels	Zhang et al., 2010
	Milroy disease (human)	
*Gata2*	Small lymph sacs; defective migration of LEC progenitors from the JV; enlarged lymph sacs and lymphatic vessels; abnormal formation of lymphovenous valve leaflets	Kazenwadel et al., 2015; Frye et al., 2018
	Emberger syndrome (human)	
*Uqcrq*	Enlarged lymph sacs; absence of lymphatic vessels; defective migration of LEC progenitors from the JV to the skin	Ma et al., 2021
*Ccbe1*	Absence of lymphatic vessels; defective migration of LEC progenitors from the JV	Zou et al., 2013
	Hennekam syndrome (human)	
*Adamts3*	Absence of lymphatic vessels	Janssen et al., 2016
*Hras*	(Gain-of-function) Dilated and hyperplastic lymphatic vessels	Ichise et al., 2010
	Costello syndrome (human)	
*Raf1*	(Gain-of-function) Enlarged lymph sacs and lymphatic vessels; increased LEC progenitors in and around the JV	Deng et al., 2013
	Noonan syndrome (human)	
*Spred1*; *Spred2*	Dilated and blood-filled lymphatic vessels	Taniguchi et al., 2007
*Itgb1*	Small lymph sacs with reduced number of LECs; absence of lymphatic vessels	Planas-Paz et al., 2012
*Ilk*	Enlarged lymph sacs with enhanced LEC proliferation	Urner et al., 2019
*Notch1*	Enlarged lymph sacs and lymphatic vessels; increased number of LEC progenitors in and around the JV (Gain-of-function) Absence of lymph sacs; reduced number of LEC progenitors around the JV	Murtomaki et al., 2012
*Adm*	Small lymph sacs with reduced LEC proliferation	Fritz-Six et al., 2008
*Calcrl*	Small lymph sacs with reduced LEC proliferation; dilated lymphatic vessels	Fritz-Six et al., 2008; Mackie et al., 2018
*Ramp2*	Small lymph sacs with reduced LEC proliferation	Fritz-Six et al., 2008; Kechele et al., 2016
*Ackr3*	Enlarged and blood-filled lymph sacs; dilated lymphatic vessels with decreased branching	Klein et al., 2014
*Foxc1*	Enlarged lymph sacs with enhanced LEC proliferation; dilated lymphatic vessels	Fatima et al., 2016
*Foxc2*	Enlarged lymph sacs with enhanced LEC proliferation; dilated lymphatic vessels; defective lymphovenous valve formation	Fatima et al., 2016; Geng et al., 2016
	Lymphedema-distichiasis syndrome (human)	
*Vezf1*	Lymphatic hypervascularization in the jugular region	Kuhnert et al., 2005
*Tie1*	Abnormally patterned lymph sacs	D’Amico et al., 2010
*Arf6*	Enlarged lymph sacs; dilated lymphatic vessels with decreased branching; delayed elongation of lymphatic vessels	Lin et al., 2017
*Sptlc2*	Enlarged lymph sacs and lymphatic vessels	Wang et al., 2021
*Pkd1*	Enlarged and blood-filled lymph sacs; dilated lymphatic vessels with decreased branching and increased number of LECs; disruption of LEC polarization	Ahrabi et al., 2010; Coxam et al., 2014; Outeda et al., 2014
*Pkd2*	Small lymph sacs; dilated lymphatic vessels with decreased branching; disruption of LEC polarization	Outeda et al., 2014
*Fat4*	Dilated lymphatic vessels with decreased branching; disruption of LEC polarization	Betterman et al., 2020
*Tgfbr1*; *Tgfbr2*	Blood-filled lymph sacs; dilated lymphatic vessels with decreased branching and enhanced LEC proliferation	James et al., 2013
*Ppp1r13b*	Dilated lymphatic vessels; numerous isolated lymphatic islands	Hirashima et al., 2008
*Wnt5a*	Dilated and blood-filled lymphatic vessels; more isolated lymphatic cysts	Lutze et al., 2019
*Cdh5*	Dilated lymph vessels with increased number of LECs; defective lymphovenous valve formation	Hägerling et al., 2018; Yang et al., 2019
*Rap1a*; *Rap1b*	Enlarged and blood-filled lymph sacs with disruption of LEC junctions; reduced density of lymphatic vessels	Xu et al., 2018
*Afdn*	Disruption of LEC junctions	Majima et al., 2013
*Cxadr*	Dilated and blood-filled lymphatic vessels; disruption of LEC junctions	Mirza et al., 2012
*Lpar4*	Enlarged lymph sacs and lymphatic vessels	Sumida et al., 2010
*Svep1*	Abnormal EC association at lymphovenous valve formation site; decreased Foxc2 expression in LECs	Karpanen et al., 2017; Morooka et al., 2017
*Ctnnb*	Enlarged lymph sacs; dilated lymphatic vessels with decreased branching; delayed elongation of lymphatic vessels; defective lymphovenous valve formation	Cha et al., 2016
*Ephb4*	Dilated, blood-filled and tortuous lymphatic vessels; abnormal formation of lymphovenous valve leaflets	Martin-Almedina et al., 2016
*Cyp26b1*	Enlarged and blood-filled lymph sacs; dilated lymphatic vessels with decreased branching; abnormal formation of lymphovenous valve leaflets	Bowles et al., 2014
*Clec1b*	Dilated, tortuous and blood-filled lymphatic vessels; lack of thrombus formation at lymphovenous valves	Suzuki-Inoue et al., 2010; Hess et al., 2014
*Syk*	Blood-filled lymphatic vessels	Abtahian et al., 2003; Finney et al., 2012
*Lcp2*	Blood-filled lymph sacs and lymphatic vessels	Abtahian et al., 2003; Bertozzi et al., 2010
*Chd4*	Enlarged and blood-filled lymph sacs and lymphatic vessels; lack of thrombus formation at lymphovenous valves	Crosswhite et al., 2016

LEC, lymphatic endothelial cell; JV, jugular vein.

### Specification of lymphatic endothelial cell progenitors

**FIGURE 2:**
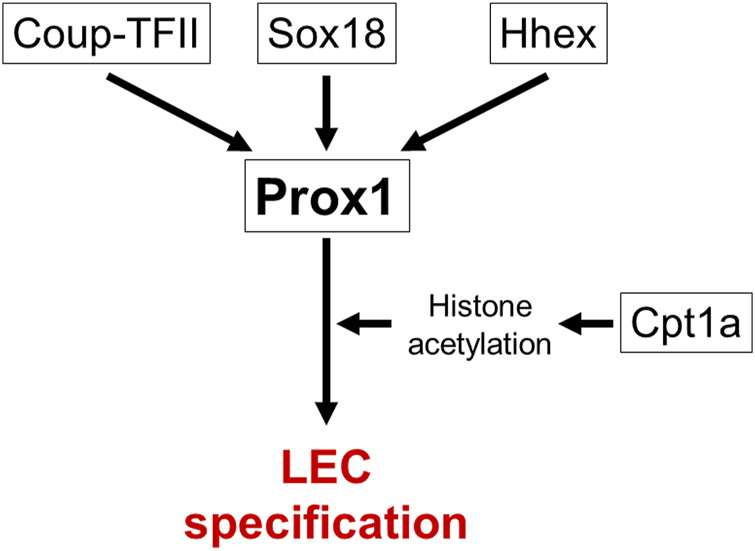
Transcriptional networks in specification of LEC progenitors. *Prox1* is a master transcriptional regulator gene of LEC specification. Several transcription factors (Sox18, Coup-TFII, Hhex) and Cpt1a-modulated histone acetylation regulate *Prox1* expression. Causative genes for fetal nuchal edema in mice are surrounded by square.

It is the first step in lymphatic vascular development that the specification of lymphatic endothelial cell (LEC) progenitors from a subpopulation of ECs in the jugular vein (JV) around embryonic day (E) 9.5 (Wigle and Oliver, 1999; Srinivasan et al., 2007; Tammela and Alitalo, 2010; Yang and Oliver, 2014). Gene mutations which impair specification of LEC progenitors cause fetal nuchal edema with hypoplastic lymphatic vessels in mice. Prospero-related homeobox 1 (*Prox1*) gene encodes a master transcriptional regulator to induce LEC progenitor differentiation and maintain LEC identity (Hong et al., 2002; Tammela and Alitalo, 2010) (Figure 2). Both loss- and gain- of function of Prox1 induce fetal nuchal edema, indicating that appropriate regulation of *Prox1* expression is essential for maintenance of lymphatic vascular morphology and fluid homeostasis (Wigle and Oliver, 1999; Harvey et al., 2005; Kim et al., 2013). *Prox1* expression is regulated by several transcription factors such as SRY-box transcription factor 18 (Sox18), chicken ovalbumin upstream promoter transcription factor 2 (Coup-TFII) and hematopoietically-expressed homeobox (Hhex) (François et al., 2008; Lin et al., 2010; Srinivasan et al., 2010; Gauvrit et al., 2018). EC-specific deletion of *Sox18*, *Nr2f2*, or *Hhex* gene in mice reduces LEC progenitors and induces fetal nuchal edema (François et al., 2008; Lin et al., 2010; Srinivasan et al., 2010; Gauvrit et al., 2018). Carnitine palmitoyl transferase 1a (Cpt1a) is an enzyme implicated in fatty acid β-oxidation-dependent synthesis of acetyl coenzyme A required for histone acetylation in the regulatory region of Prox1-target genes (Wong et al., 2017; Schlaepfer and Joshi, 2020). LEC-specific deletion of *Cpt1a* in mice induces fetal nuchal edema (Wong et al., 2017). Wingless type MMTV integration site family, member 5b (Wnt5b) upregulates *Prox1* expression in zebrafish and human embryonic stem cell-derived angioblasts (Nicenboim et al., 2015). It will be intriguing whether *Wnt5b* mutation induces fetal nuchal edema in mice.

### Migration and proliferation of lymphatic endothelial cell progenitors

**FIGURE 3:**
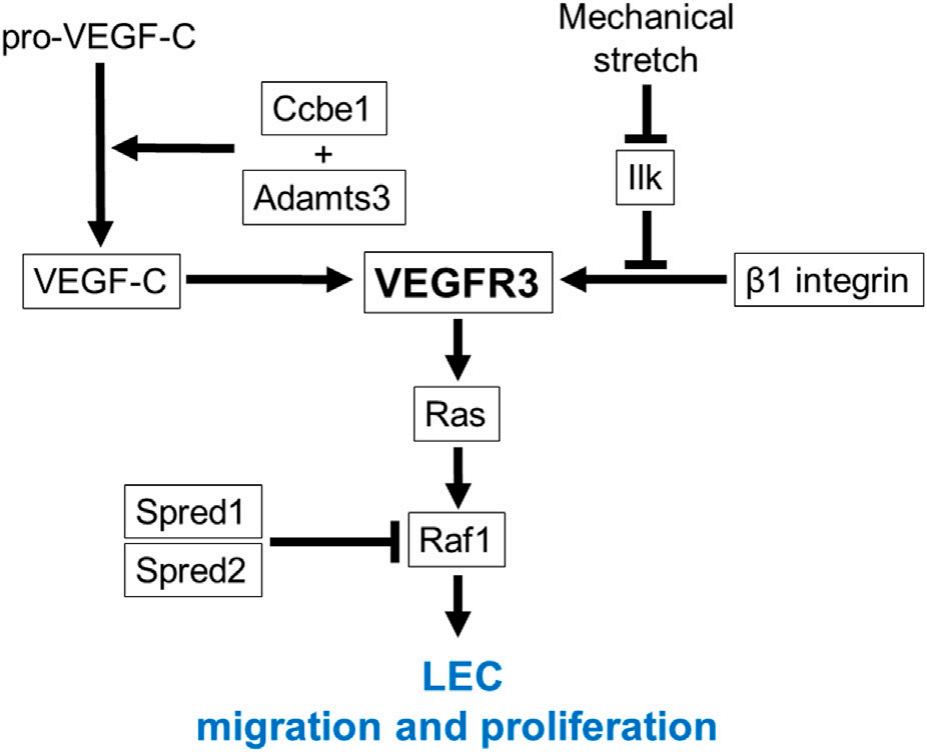
VEGFR3 signaling in migration and proliferation of LEC progenitors. VEGFR3 functions as a main signaling receptor for migration and proliferation of LEC progenitors. Ccbe1 and Adamts3 cleave pro-VEGF-C and produce active form that allows binding to VEGFR3. Binding of VEGF-C to VEGFR3 results in tyrosine phosphorylation, which in turn activates Ras/Raf signaling. Raf activity is negatively regulated by Spred1 and Spred2. VEGFR3 signaling is also regulated by β1 integrin in a VEGF-C independent manner. β1 integrin interaction to VEGFR3 is inhibited by Ilk, but upon mechanical stretch, the complex of β1 integrin and Ilk is transiently disrupted, which in turn promotes VEGFR3 phosphorylation. Causative genes for fetal nuchal edema in mice are surrounded by square.

Differentiated LEC progenitors migrate away from the JV toward the neighboring mesenchymal tissues to form early lymph sacs (Wigle and Oliver, 1999; Tammela and Alitalo, 2010; Yang et al., 2012; Yang and Oliver, 2014). Gene mutations which impair migration and proliferation of LEC progenitors cause fetal nuchal edema with hypoplastic lymphatic vessels in mice. Vascular endothelial growth factor receptor 3 (VEGFR3/Flt4) is induced in LEC progenitors with elevated *Prox1* expression (Karkkainen et al., 2004). VEGF-C upregulates *Prox1* expression levels *via* Prox1-VEGFR3 feedback loop, which maintains the number of LEC progenitors (Srinivasan et al., 2014). Migration and proliferation of LEC progenitors are promoted by intracellular signaling *via* VEGFR3 and its ligand VEGF-C produced by mesenchymal cells (Karkkainen et al., 2004) (Figure 3). *Vegfc* knockout mice exhibit fetal nuchal edema with hypoplastic lymphatic vessels due to impaired migration of LEC progenitors from JV (Karkkainen et al., 2004). *Chy* mice, which carries a heterozygous inactivating point mutation in tyrosine kinase domain of VEGFR3 induced by ethylnitrosourea, exhibit fetal nuchal edema with lack of lymphatic vessels (Zhang et al., 2010). Flt4 expression levels are also important for lymphatic vascular development. GATA2 regulates VEGFR3 expression in LECs by directly binding to the intronic enhancer region of the *FLT4* gene (Frye et al., 2018). Mitochondrial respiratory chain complex III QPC subunit (Uqcrq) is required for the maintenance of epigenetic modifications at the *FLT4* promoter (Ma et al., 2021). Deletion of *Gata2* or *Uqcrq* in mice induces fetal nuchal edema with defective migration of LEC progenitors from the JV (Frye et al., 2018; Ma et al., 2021). VEGF-C, originally generated as a prepropeptide, requires proteolysis to produce active form that allows binding to VEGFR3 (Joukov et al., 1997). Collagen and calcium-binding EGF domain-containing protein 1 (Ccbe1) and a disintegrin and metalloproteinase with thrombospondin motifs 3 (Adamts3) play a critical role in proteolytic cleavage of pro-VEGF-C (Jeltsch et al., 2014). Deletion of *Ccbe1* or *Adamts3* in mice induces fetal nuchal edema with lack of lymphatic vessels (Zou et al., 2013; Janssen et al., 2016). Binding of VEGF-C to VEGFR3 results in tyrosine phosphorylation, which in turn activates Ras/Raf/extracellular signal regulated kinase (ERK) signaling and eventually promotes LEC migration and sprouting (Mäkinen et al., 2021). In humans, gain-of-function mutations in Ras pathway-related genes including *HRAS*, *RAF1*, *PTPN11*, *KRAS*, *SOS1* and *RIT1* are actually identified in Noonan syndrome and related disorder (Costello syndrome) patients associated with increased NT (Croonen et al., 2013; Sleutjes et al., 2022). Noonan syndrome is a multiple malformation syndrome with characteristic facies, congenital heart disease, and short stature, and presents with prenatal and postnatal lymphedema (Noonan, 2006; Sleutjes et al., 2022). Some of these gene mutations have been shown to cause fetal nuchal edema in mouse models. EC-specific overexpression of *Hras* in mice induces fetal nuchal edema with dilated and hyperplastic lymphatic vessels (Ichise et al., 2010). Raf1 is phosphorylated at Ser259 downstream of phosphatidylinositol 3 kinase (PI3K)/Akt signaling and is inhibited under normal condition in ECs (Ren et al., 2010). Thus, substitution of this Serine to Phenylalanine (S259F) or to Threonine (S259T) results in gain-of-function mutation of Raf1 (Pandit et al., 2007; Ko et al., 2008). EC-specific *RAF1* ^S259A^ expression in mice causes fetal nuchal edema with increased number of LEC progenitors around the JV (Deng et al., 2013). Raf activity is also negatively regulated by Sprouty-related Ena/VASP homology1-domain containing (Spred) (Wakioka et al., 2001). *Spred1* and *Spread2* double knockout mice exhibit fetal nuchal edema with blood-filled lymphatic vessels (Taniguchi et al., 2007). VEGFR3 signaling is also activated by β1 integrin in a VEGF-C-independent manner (Planas-Paz et al., 2012). It has been proposed that β1 integrin interaction to VEGFR3 is inhibited by integrin-linked kinase (Ilk), but upon mechanical stretch, the complex of β1 integrin and Ilk is transiently disrupted, which in turn promotes VEGFR3 phosphorylation and subsequent LEC proliferation (Urner et al., 2019). EC-specific deletion of *Itgb1* in mice induces fetal nuchal edema with hypoplastic lymph sacs and lymphatic vessels and reduced LEC proliferation (Planas-Paz et al., 2012). LEC-specific deletion of *Ilk* in mice induces fetal nuchal edema with enhanced LEC proliferation (Urner et al., 2019). Notch1 is also suggested as an inhibitory molecule for VEGFR3 signaling and LEC migration (Zheng et al., 2011; Choi et al., 2017). Loss-of-function mutation of Notch1 in LECs induces fetal nuchal edema with increased number of LEC progenitors around the JV (Murtomaki et al., 2012). On the other hand, gain-of-function mutation of Notch1 in LECs induces fetal nuchal edema with lymph sac hypoplasia and downregulation of Coup-TFII and VEGFR3 (Murtomaki et al., 2012). It is interesting to note that fetal nuchal edema is not caused by only hypoplasia but also hyperplasia of lymphatic vessels in mice.

Migrated LECs begin to form lymph sacs (Tammela and Alitalo, 2010; Yang et al., 2012; Yang and Oliver, 2014). Enlarged jugular lymph sacs are detected in some fetuses exhibiting increased NT (Bekker et al., 2005). Gene mutations which impair lymph sac formation cause fetal nuchal edema in mice. Adrenomedullin (Adm)-calcitonin receptor-like receptor (Calcrl) axis promotes lymph sac formation through enhanced LEC proliferation (Fritz-Six et al., 2008). Receptor activity-modifying proteins (Ramps) regulate the interaction between Adm and Calcrl (McLatchie et al., 1998). Knockout mice for *Adm*, *Calcr1* or *Ramp2* exhibit fetal nuchal edema with hypoplastic lymph sacs (Fritz-Six et al., 2008; Kechele et al., 2016; Mackie et al., 2018). C-X-C chemokine receptor type 7 (Cxcr7/Ackr3) is a decoy receptor for Adm and modulates Adm-mediated lymphatic vascular development. Deletion of *Ackr3* in mice induces fetal nuchal edema with enlarged lymph sacs (Klein et al., 2014). Forkhead box (Fox) transcription factor Foxc1 and Foxc2 regulate lymph sac development by modulating LEC proliferation. LEC-specific deletion of *Foxc1* or *Foxc2* in mice induces fetal nuchal edema with enlarged lymph sacs (Fatima et al., 2016). In addition, knockout mice for the *Vezf1* gene encoding vascular endothelial zinc finger 1, a transcriptional regulatory protein, exhibit fetal nuchal edema with lymphatic hypervascularization in the jugular lymph sac region (Kuhnert et al., 2005). Knockout mice for *Tie1*, a receptor tyrosine kinase that regulates migration and proliferation in ECs, exhibit fetal nuchal edema with abnormally patterned lymph sacs (D’Amico et al., 2010).

### Formation of lymphatic vascular network

As lymph sacs are formed, LECs continue to proliferate and migrate, forming lymphatic vascular network throughout the developing embryo (Tammela and Alitalo, 2010; Yang and Oliver, 2014). Gene mutations which impair the formation of lymphatic vascular network cause fetal nuchal edema in mice. ADP-ribosylation factor 6 (Arf6) is a small GTPase which regulates internalization of β1 integrin in LECs. LEC-specific deletion of *Arf6* in mice induces fetal nuchal edema with suppressed elongation of dermal lymphatic vessels to the dorsal midline (Lin et al., 2017). Serine palmitoyltransferase long chain base subunit 2 (Sptlc2), the rate-limiting enzyme of sphingolipid biosynthesis, increases Coup-TFII transcriptional activity. EC-specific deletion of *Sptlc2* in mice induces fetal nuchal edema with dilated dermal lymphatic vessels (Wang et al., 2021). Polycystin is a cell surface receptor involved in cell-cell and cell-matrix interactions and is encoded by *PKD1* and *PKD2*, the genes responsible for autosomal dominant polycystic kidney disease (Delmas, 2004). FAT4 is atypical cadherin which is expressed on the cell surface of plasma membrane (Katoh, 2012). Polycystin and FAT4 regulate polarity in LECs and deletion of *Pkd1*, *Pkd2* or *Fat4* in mice induces fetal nuchal edema with dilatation and decreased vascular branching of dermal lymphatic vessels (Ahrabi et al., 2010; Coxam et al., 2014; Outeda et al., 2014; Betterman et al., 2020). Transforming growth factor-β is widely known to play an important role in multiple cellular process (Barnard et al., 1990), and EC-specific deletion for its receptor *Tgfbr1* or *Tgfbr2* induces fetal nuchal edema with dilated dermal lymphatic vessels with decreased vascular branching (James et al., 2013). Furthermore, we previously reported that knockout mice for *Ppp1r13b*, which encodes apoptosis stimulating protein of p53 (Aspp1), exhibit fetal nuchal edema with numerous isolated lymphatic islands as well as disorganized lymphatic vessels in embryonic skin (Hirashima et al., 2008). Deletion of *Wnt5a,* a member of the WNT family in mice also induces fetal nuchal edema with isolated lymphatic cysts in embryonic skin (Lutze et al., 2019).

Gene mutations which affect LEC junctions cause fetal nuchal edema in mice. VE-cadherin is a representative endothelial cell-cell adhesion molecule responsible for vascular integrity (Zhang et al., 2020). LEC-specific knockout mice for *Cdh5* gene encoding VE-cadherin exhibit fetal nuchal edema with dilated lymphatic vessels (Hägerling et al., 2018). LEC-specific knockout mice for *Rap1a/b* exhibit fetal nuchal edema with enlarged and blood-filled lymph sacs accompanied by destabilization of VE-cadherin-mediated LEC junctions (Xu et al., 2018). Afadin (*Afdn*) is a membrane scaffold protein with filamentous-actin binding activity and is colocalized with VE-cadherin at LEC junctions. EC-specific deletion of *Afdn* in mice induces fetal nuchal edema with reduced VE-cadherin expression and numerous punctures in lymphatic endothelium (Majima et al., 2013). The coxsackie- and adenovirus receptor (CAR) is a cell adhesion molecule localized at LEC junctions. Deletion of the *Cxadr* gene encoding CAR in mice induces fetal nuchal edema with enlarged blood-filled lymphatic vessels (Mirza et al., 2012).

### Formation of lymphovenous valves

The downstream end of the thoracic duct is connected to the vein at the jugular venous angle, which allows the lymph flows into the blood circulation. Lymphovenous valves formed at the connection prevent the influx of blood cells into the lymphatic vessels (Yang and Oliver, 2014). Gene mutations which impair formation of lymphovenous valves cause fetal nuchal edema in mice. Lymphovenous valve formation begins with the differentiation of a partial venous ECs into lymphovenous valve ECs (LVV-ECs) (Geng et al., 2016). LVV-ECs highly expresses Gata2 and Foxc2, which play critical roles in formation of lymphovenous valves (Yang and Oliver, 2014; Geng et al., 2016). LEC-specific deletion of *Gata2* in mice induces fetal nuchal edema with blood-filled lymph sacs due to abnormal formation of lymphovenous valve leaflets (Kazenwadel et al., 2015). Approximately 50% of heterozygous knockout mice for *Foxc2* exhibit severe fetal nuchal edema with lack of lymphovenous valves and enlarged lymph sacs (Geng et al., 2016). The similar phenotypes are reported in knockout mice for the *Lpa4* gene encoding lysophosphatidic acid receptor 4 (Sumida et al., 2010). Extracellular matrix protein Svep1 regulates *Foxc2* expression in LECs. LEC-specific deletion of *Svep1* in mice induces severe fetal nuchal edema (Karpanen et al., 2017; Morooka et al., 2017). VE-cadherin encoded by the *Cdh5* gene is the mechanotransduction protein which senses shear stress and elevates *FOXC2* expression (Yang et al., 2019). β-catenin (*Ctnnb*), an intracellular protein that colocalizes with VE-cadherin, mediates shear stress-induced upregulation of *FOXC2* (Cha et al., 2016). LEC-specific deletion of *Cdh5* or *Ctnnb* in mice induces fetal nuchal edema with lack of lymphovenous valves (Cha et al., 2016; Yang et al., 2019). Intracellular signaling mediated by the receptor tyrosine kinase EphB4 and its ligand EphrinB2 is also required for formation of lymphovenous valves. LEC-specific knockout mice for *Ephb4* exhibit fetal nuchal edema with blood-filled lymphatic vessels due to hypoplasia of lymphovenous valve leaflets (Martin-Almedina et al., 2016). Retinoic acid is a regulator of lymphovenous valve formation. Deletion of cytochrome P450 26B1 (*Cyp26b1*), which catalyzes the degradation of retinoic acid, induces fetal nuchal edema with blood-filled lymph sacs due to expanded size of lymphovenous valve leaflets (Bowles et al., 2014).

LECs at lymphovenous valves can come in direct contact with blood cells if influx of blood cells into lymphatic vessels occurs. LEC-mediated platelet activation and thrombus formation at lymphovenous valves has been implicated in lymph-blood partitioning (Welsh et al., 2016). Gene mutations which impair platelet activation cause fetal nuchal edema in mice. Podoplanin expressed on LECs activates the C-type lectin-like receptor 2 (Clec2/Clec1b) in platelets, leading to platelet activation *via* Syk-Slp76/Lcp2 signaling (Welsh et al., 2016). Knockout mice for *Clec1b*, *Syk* or *Lcp*2 encoding these signaling molecules exhibit fetal nuchal edema with blood-filled lymphatic vessels (Abtahian et al., 2003; Bertozzi et al., 2010; Suzuki-Inoue et al., 2010; Finney et al., 2012; Hess et al., 2014). Besides, LEC-specific deletion of chromodomain helicase DNA binding protein 4 (*Chd4*), chromatin remodeling enzyme induces fetal nuchal edema with blood-filled lymphatic vessels due to lack of thrombus formation at lymphovenous valves by increased plasmin activity (Crosswhite et al., 2016).

## Cardiac anomaly

Cardiac anomalies or reduction in cardiac contractility decreases cardiac output, which causes an elevation in venous pressure and a subsequent elevation in capillary hydrostatic pressure. Edema occurs when fluid leakage by high capillary hydrostatic pressure exceeds the ability of the lymphatic system to return fluid to the blood circulation (Cho and Atwood, 2002). The prevalence of cardiac anomalies correlates with NT thickness in fetuses (Atzei et al., 2005; Souka et al., 2005). Gene mutations which induce cardiac anomalies cause fetal nuchal edema (Table 2).

**TABLE 2:** List of genes and mutant phenotypes related to fetal nuchal edema with cardiac anomalies in mice (loss-of-function mutations).

Gene	Mutant phenotype	References
*Gata4; Gata6*	VSD; PTA; thin ventricular myocardium	Xin et al., 2006
	Congenital heart diseases (human)	
*Tbx1*	VSD; PTA	Vitelli et al., 2002; Burger et al., 2016
	Congenital heart diseases (human)	
*Dock1*	VSD; DORV	Sanematsu et al., 2010
*Adm*	Small ventricular chamber size; thin and convoluted ventricular myocardium	Caron and Smithies, 2001
*Calcrl*	Small heart; thin and convoluted ventricular myocardium	Dackor et al., 2006
*Nfat5*	Thin ventricular myocardium	Mak et al., 2011
*Chd7*	VSD	Bosman et al., 2005
	CHARGE syndrome (human)	
*Crk*	VSD; DORV; thin ventricular myocardium and interventricular septum; dilated ventricular chamber	Park et al., 2006; Imamoto et al., 2020
*Fkbp1a*	VSD; enlarged heart; thin ventricular myocardium	Shou et al., 1998
*Myh10*	VSD; DORV	Ma and Adelstein, 2014
*Ngly1*	VSD	Fujihira et al., 2017
*Sp3*	AVSD; DORV; thin ventricular myocardium	van Loo et al., 2007
*Strn3*	VSD	Dickinson et al., 2016

AVSD, atrioventricular septal defect; DORV, double outlet right ventricle; PTA, persistent truncus arteriosus; VSD, ventricular septal defect.

Congenital heart disease is the leading cause of early postnatal mortality (Soares, 2018). Therefore, in fetuses with increased NT due to cardiac anomalies, it may have a significant impact on prenatal as well as postnatal pathophysiology. Early diagnosis of the presence or absence of cardiac anomalies in fetuses with increased NT is very important.

As described in several review articles on cardiac development, the mature four-chambered heart is formed from a liner heart tube through a complex series of events, including rightward looping, cushion formation, cardiac chamber septation and outflow tract septation (Srivastava and Olson, 2000; Paige et al., 2015; Desgrange et al., 2018). A number of gene mutations have been reported to cause cardiac anomalies in mice (Bruneau, 2002; Desgrange et al., 2018), but only a few of them have been implicated in fetal nuchal edema. Gene mutations which impair these developmental processes cause fetal nuchal edema in mice. *GATA4* regulates all the processes of cardiac development (Paige et al., 2015). T-box transcription factor 1 (*TBX1*) regulates the later process such as chamber septation (Paige et al., 2015). Knockout mice for *Tbx1* or double heterozygous knockout mice for *Gata4* and *Gata6* exhibit fetal nuchal edema with ventricular septal defect (VSD) and persistent truncus arteriosus (PTA) (Vitelli et al., 2002; Xin et al., 2006; Burger et al., 2016). Loss-of-function mutation in the *Dock1* gene encoding an atypical Rac activator causes fetal nuchal edema with VSD and double outlet right ventricle (DORV) (Sanematsu et al., 2010). Several other genes have been reported to be associated with cardiac anomalies including VSD and DORV (Table 2).

Reduction in cardiac contractility is associated with myocardial wall thinning (Maciver, 2011). Gene mutations which impair cardiomyocyte proliferation even with normal compartmentalization of four chambers and the great arteries cause fetal nuchal edema in mice. Deletion of *Adm*, *Calcrl* or nuclear factor of activated T-cells 5 (*Nfat5*) in mice reduces cardiomyocyte proliferation and induces thinning of compact zone of ventricular myocardium (Caron and Smithies, 2001; Dackor et al., 2006; Mak et al., 2011).

Despite the high prevalence of cardiac anomalies in patients with increased NT, much fewer number of gene mutations responsible for cardiac anomalies have been reported, compared to those for defective lymphatic vascular development. Kalisch-Smith et al. (2021) reported that mice fed with iron-deficient diets exhibit fetal nuchal edema with VSD and DORV. Taken together, a significant percentage of cardiac anomaly cases may be due to chromosomal abnormalities or non-genetic factors such as nutritional condition of embryos.

## Others

### Anemia

Anemia in human fetuses has been implicated in increased NT (Tercanli et al., 2001; Souka et al., 2005). Blood type incompatibility between a mother and fetus is known to cause hemolysis, jaundice, and severe anemia in the fetus (Prefumo et al., 2019). Severe anemia induces high-output heart failure, leading to severe edema called as hydrops fetalis (Tongsong et al., 2010). Human parvovirus B19 infection causes increased NT with anemia by inducing apoptosis of erythroid precursors in liver (Poole et al., 2004; Giorgio et al., 2010). Gene mutation which induces anemia causes fetal nuchal edema in mice (Table 3).

**TABLE 3:** List of genes and mutant phenotypes related to fetal nuchal edema with anemia or blood vascular endothelial barrier anomalies in mice (loss-of-function mutations if not stated).

Gene	Mutant phenotype	References
*Stk40*	Anemia; small liver with increased cell apoptosis	Wang et al., 2017
*Flt1*	Enhanced vascular permeability	Otowa et al., 2016
*Myc*	(Gain-of-function) Disruption of BEC junctions	Kokai et al., 2009
*Enah; Evl; Vasp*	Disruption of BEC junctions	Furman et al., 2007

BEC, blood vascular endothelial cell.

Serine/threonine kinase 40 (*Stk40*) knockout mice exhibit fetal nuchal edema with reduced hematocrits and hemoglobin levels (Wang et al., 2017). In this mutant, a primary defect appears enhanced cell apoptosis in the liver, the hematopoietic organ during fetal life (Lewis et al., 2021). Thus, gene mutations affecting hepatocyte proliferation and function during development can be a possible cause of nuchal edema.

### Blood vascular endothelial barrier anomaly

Fetal nuchal edema is remarkably observed after E13.5 in mice (D’Amico et al., 2010; Lin et al., 2017), while defective blood vascular development often results in embryonic lethality before E12.5 (Shalaby et al., 1995; Jeansson et al., 2011). Therefore, it is unlikely that the gene mutations that causes major defects in blood vascular development are associated with fetal nuchal edema. On the other hand, gene mutations which impair blood vascular endothelial barrier cause fetal nuchal edema in mice (Table 3). We previously reported that heterozygosity in mice for the *Flt1* gene encoding VEGFR1, a decoy receptor for VEGF-A, causes fetal nuchal edema. This mouse model exhibits an enhanced phosphorylation of VEGFR2, the main signaling receptor for VEGF-A, and an increased vascular permeability without affecting vascular morphology (Otowa et al., 2016). EC-specific overexpression of *Myc* in mice causes fetal nuchal edema by inducing the apoptosis of blood vascular ECs (BECs) and disruption of vascular endothelial barrier (Kokai et al., 2009). Enabled/vasodilator (Ena/VASP), expresses both at focal adhesions and at cell-cell junctions, contributes to stabilization of cell adhesion. Ena/VASP-deficient mice (triple knockout mice for *Enah*, *Vasp* and *Evl*) exhibit fetal nuchal edema with gap formation between BECs in venules (Furman et al., 2007). These reports in mice indicate that increased fluid leakage from blood vessels due to disruption of vascular integrity may also be a cause of increased NT in human cases.

## Conclusion

In this review, we described a number of gene mutations, which cause defective lymphatic vascular development, cardiac anomalies, anemia and blood vascular endothelial barrier anomalies, are associated with fetal nuchal edema. Therefore, the gene mutations which induce increased NT are expected to be diverse and vary for individual cases. In the field of oncology, gene panel testing is performed for comprehensive evaluation of numerous cancer-related gene mutations, leading to appropriate treatment for individual patients (Reid and Pal, 2020; Pereira et al., 2021). Although gene panel testing may also be useful for the diagnosis of increased NT, further identification of the causative genes is required to achieve it. Recently, studies of exome sequencing data analysis have been performed for diagnostic approach to human fetuses with increased NT, resulting in the listing of some candidate genes (Mellis et al., 2022; Pauta et al., 2022). Studies using gene mutant mice could be very useful to examine whether these candidate genes are causative genes for fetal nuchal edema, leading to the identification of novel causative genes and the realization of gene panel testing.

Although increased NT is considered to be a transient physiological finding (Yoshida et al., 2008), lymphatic dysfunction and heart defects described in this review are supposed to remain after birth. Even a subtle change in vascular permeability may put an impact on health during the long lifespan. In fact, *Prox1* haploinsufficient mice, which survive to adulthood despite anatomical and functional lymphatic anomalies, exhibit obesity, an underlying risk for metabolic syndrome (Harvey et al., 2005). Prediction of the risk of postnatal disease onset by gene panel testing for fetal nuchal edema is expected to contribute to the prevention of various diseases and may present new possibilities for prenatal diagnosis. Taken together, studies of fetal nuchal edema using gene mutant mice will open up new avenues for the accurate diagnosis and treatment of increased NT in clinical medicine.
